# 2-[3-(2-Pyrid­yl)pyrazin-2-yl]pyridinium tetra­chloridoaurate(III)

**DOI:** 10.1107/S1600536809012264

**Published:** 2009-04-08

**Authors:** Sema Öztürk Yıldırım, Mehmet Akkurt, Nasser Safari, Vahid Amani, Vickie McKee

**Affiliations:** a Department of Physics, Faculty of Arts and Sciences, Erciyes University, 38039 Kayseri, Turkey; b Department of Chemistry, Shahid Beheshti University, GC, Evin, Tehran 1983963113, Iran; c Chemistry Department, Loughborough University, Loughborough Leics LE11 3TU, England

## Abstract

In the anion of the title compound, (C_14_H_11_N_4_)[AuCl_4_], the Au^III^ atom has an almost perfect square-planar coordination. In the crystal structure, an intra­molecular N—H⋯N and intermolecular C—H⋯Cl hydrogen bonds are observed. In addition, there is also a ring–metal inter­action between the pyrazine ring and the Au^III^ atom; the distance between the centroid of the pyrazine ring and the Au^III^ atom is 3.628 (2) Å.

## Related literature

For proton-transfer systems involving [AuCl_4_], see: Calleja *et al.* (2001 ▶); Hasan *et al.* (1999 ▶); Hojjat Kashani *et al.* (2008 ▶); Johnson & Steed (1998 ▶); Safari *et al.* (2009 ▶); Yap *et al.* (1995 ▶); Zhang *et al.* (2006 ▶).
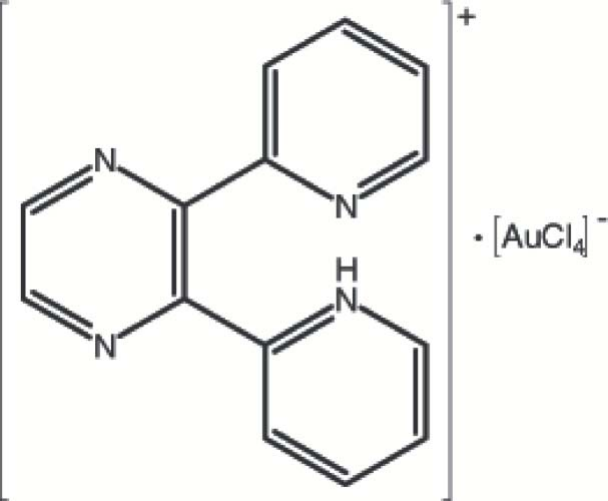


## Experimental

### 

#### Crystal data

(C_14_H_11_N_4_)[AuCl_4_]
*M* *_r_* = 574.04
Monoclinic, 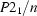

*a* = 7.4098 (6) Å
*b* = 15.5188 (13) Å
*c* = 14.6197 (12) Å
β = 90.380 (1)°
*V* = 1681.1 (2) Å^3^
*Z* = 4
Mo *K*α radiation
μ = 9.39 mm^−1^
*T* = 150 K
0.19 × 0.14 × 0.09 mm

#### Data collection

Bruker APEXII CCD diffractometer
Absorption correction: multi-scan (**SADABS**; Sheldrick, 2003 ▶) *T*_min_ = 0.238, *T*_max_ = 0.430
19688 measured reflections
5261 independent reflections
4363 reflections with *I* > 2σ(*I*)
*R*_int_ = 0.034

#### Refinement

*R*[*F* ^2^ > 2σ(*F* ^2^)] = 0.024
*wR*(*F* ^2^) = 0.052
*S* = 0.98
5261 reflections
208 parameters
H-atom parameters constrained
Δρ_max_ = 1.19 e Å^−3^
Δρ_min_ = −0.59 e Å^−3^

### 

Data collection: *APEX2* (Bruker, 2005 ▶); cell refinement: *SAINT* (Bruker, 2005 ▶); data reduction: *SAINT*; program(s) used to solve structure: *SIR97* (Altomare *et al.*, 1999 ▶); program(s) used to refine structure: *SHELXL97* (Sheldrick, 2008 ▶); molecular graphics: *ORTEP-3 for Windows* (Farrugia, 1997 ▶); software used to prepare material for publication: *WinGX* (Farrugia, 1999 ▶).

## Supplementary Material

Crystal structure: contains datablocks global, I. DOI: 10.1107/S1600536809012264/is2406sup1.cif

Structure factors: contains datablocks I. DOI: 10.1107/S1600536809012264/is2406Isup2.hkl

Additional supplementary materials:  crystallographic information; 3D view; checkCIF report

## Figures and Tables

**Table:** 

Au1—Cl1	2.2801 (8)
Au1—Cl2	2.2725 (8)
Au1—Cl3	2.2818 (8)
Au1—Cl4	2.2805 (8)

**Table:** 

Cl3—Au1—Cl4	89.48 (3)
Cl1—Au1—Cl4	90.52 (3)
Cl1—Au1—Cl2	90.14 (3)
Cl1—Au1—Cl3	178.97 (3)
Cl2—Au1—Cl3	89.87 (3)
Cl2—Au1—Cl4	179.25 (3)

**Table 2:** Hydrogen-bond geometry (Å, °)

*D*—H⋯*A*	*D*—H	H⋯*A*	*D*⋯*A*	*D*—H⋯*A*
N3—H3⋯N4	0.86	1.71	2.540 (3)	160
C9—H9⋯Cl3	0.93	2.81	3.699 (3)	161
C11—H11⋯Cl4^i^	0.93	2.83	3.497 (3)	130

Symmetry code: (i) 
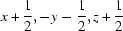
.

**Fig. 1.**
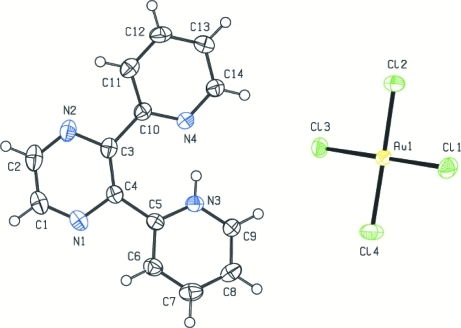
ORTEP drawing of the title compound with the atom numbering scheme. Displacement ellipsoids for non-H atoms are drawn at the 50% probability level.

**Fig. 2.**
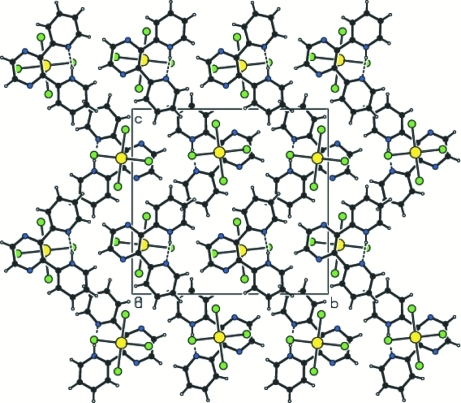
The packing and hydrogen bonding interactions of the title compound, viewed down a-axis.

**Fig. 3.**
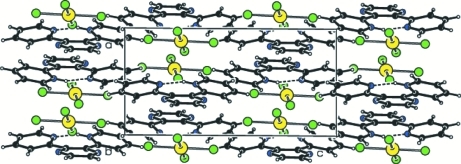
The packing and hydrogen bonding interactions of the title compound, viewed down b-axis.

**Fig. 4.**
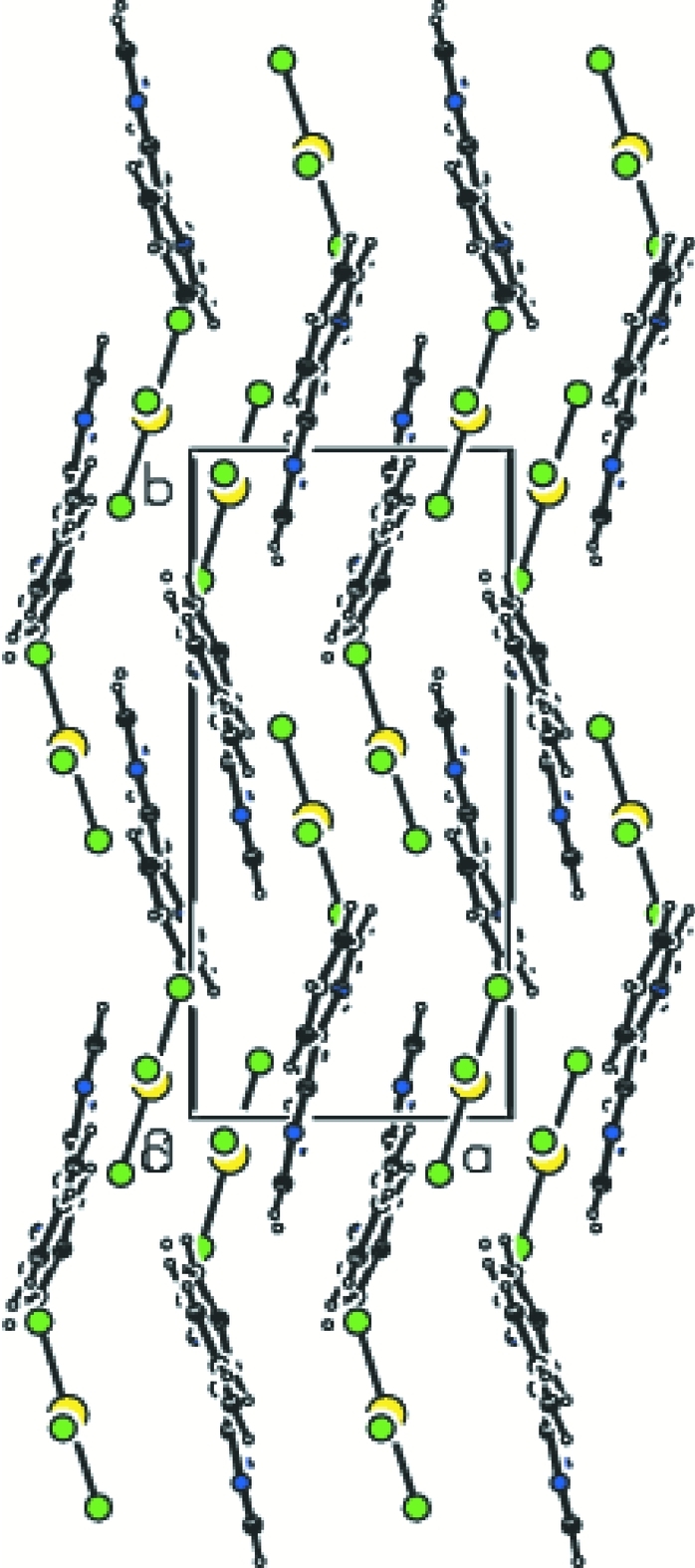
The packing and hydrogen bonding interactions of the title compound, viewed down c-axis.

## supplementary crystallographic information

### Comment

There are several proton transfer systems using HAuCl_4_ with proton acceptor molecules, such as [EMI][AuCl_4_] and [BMI]_2_[AuCl_4_].2H_2_O (Hasan *et al.*, 1999), [H_2_bipy][AuCl_4_][Cl] (Zhang *et al.*, 2006), [H_7_O_3_][15-crown-5][AuCl_4_] and [H_5_O_2_][benzo-15-crown-5]_2_[AuCl_4_] (Johnson & Steed, 1998), [H_5_O_2_]_2_[12-crown-4]_2_[AuCl_4_]_2_, [H_3_O][18-crown-6][AuCl_4_] and [H_3_O][4-nitrobenzo-18-crown-6][AuCl_4_] (Calleja *et al.*, 2001), [DPpy.H][AuCl_4_] (Yap *et al.*, 1995), [H_2_DA18C6][AuCl_4_].2H_2_O (Hojjat Kashani *et al.*, 2008) and [dafonium][dafone][AuCl_4_] (Safari *et al.*, 2009), where EMI is 1-ethyl-3-methylimidazolium, BMI is 1-butyl-3-methylimidazolium, H_2_bipy is 2, 2'-bipyridinium, DPpy.H is 2,6-diphenylpyridinium, H_2_DA18C6 is 1,10-diazonia-18-crown-6, dafonium is 9-oxo-4,5-diazafluoren-4-ium and dafone is 4,5-diazafluoren-9-one, have been synthesized and characterized by single-crystal X-ray diffraction methods. We report herein the synthesis and crystal structure of the title compound.

In the anion of the title compound (Fig. 1), the Au^III^ ion has a square-planar coordination. In the anion, the Au—Cl bond lengths and angles (Table 1) are within normal ranges.

In the crystal structure, inter- and intramolecular C—H···Cl hydrogen bonding interactions (Table 2) link the molecules. Furthermore, it is also observed a ring-metal interaction between the centroid of the pyrazine ring (N1/N2/C1–C4) and the atom Au1 (5/2 - *x*, -1/2 + *y*, 1/2 - *z*) with a distance of 3.628 (2) Å. The packing and the hydrogen bonding interactions of (I) down the *a*, *b* and *c*-axes are given in Figures 2, 3 and 4, respectively.

### Experimental

For the preparation of the title compound, a solution of 2,3-bis(2-pyridyl)pyrazine (0.13 g, 0.55 mmol) in acetonitrile (10 ml) was added to a solution of HAuCl_4_.3H_2_O, (0.21 g, 0.55 mmol) in ethanol (5 ml) and the resulting yellow solution was stirred for 15 min at 313 K. This solution was left to evaporate slowly at room temperature. After one week, yellow block crystals of the title compound were isolated (yield 0.23 g, 72.8%; m.p. 419 K).

### Refinement

All H atoms were found in a difference Fourier map. H atoms were positioned geometrically and refined using a riding model, with C—H = 0.93 Å and N—H = 0.86 Å, and with *U*_iso_(H) = 1.2*U*_eq_(C or N). The highest residual peak is located 0.79 Å from atom Au1 and the deepest hole is located 1.55 Å from atom Au1.

### Crystal data

**Table:**

(C_14_H_11_N_4_)[AuCl_4_]	*F*(000) = 1080
*M**_r_* = 574.04	*D*_x_ = 2.268 Mg m^−^^3^
Monoclinic, *P*2_1_/*n*	Mo *K*α radiation, λ = 0.71073 Å
Hall symbol: -P 2yn	Cell parameters from 6388 reflections
*a* = 7.4098 (6) Å	θ = 2.6–30.4°
*b* = 15.5188 (13) Å	µ = 9.39 mm^−^^1^
*c* = 14.6197 (12) Å	*T* = 150 K
β = 90.380 (1)°	Block, yellow
*V* = 1681.1 (2) Å^3^	0.19 × 0.14 × 0.09 mm
*Z* = 4	

### Data collection

**Table:**

Bruker APEXII CCD diffractometer	5261 independent reflections
Radiation source: sealed tube	4363 reflections with *I* > 2σ(*I*)
graphite	*R*_int_ = 0.034
φ and ω scans	θ_max_ = 31.0°, θ_min_ = 1.9°
Absorption correction: multi-scan (*SADABS*; Sheldrick, 2003)	*h* = −10→10
*T*_min_ = 0.238, *T*_max_ = 0.430	*k* = −22→21
19688 measured reflections	*l* = −21→20

### Refinement

**Table:**

Refinement on *F*^2^	Primary atom site location: structure-invariant direct methods
Least-squares matrix: full	Secondary atom site location: difference Fourier map
*R*[*F*^2^ > 2σ(*F*^2^)] = 0.024	Hydrogen site location: inferred from neighbouring sites
*wR*(*F*^2^) = 0.052	H-atom parameters constrained
*S* = 0.98	*w* = 1/[σ^2^(*F*_o_^2^) + (0.025*P*)^2^], where *P* = (*F*_o_^2^ + 2*F*_c_^2^)/3
5261 reflections	(Δ/σ)_max_ = 0.001
208 parameters	Δρ_max_ = 1.19 e Å^−^^3^
0 restraints	Δρ_min_ = −0.59 e Å^−^^3^

### Special details

**Table:**

Geometry. Bond distances, angles *etc*. have been calculated using the rounded fractional coordinates. All su's are estimated from the variances of the (full) variance-covariance matrix. The cell e.s.d.'s are taken into account in the estimation of distances, angles and torsion angles
Refinement. Refinement on *F*^2^ for ALL reflections except those flagged by the user for potential systematic errors. Weighted *R*-factors *wR* and all goodnesses of fit *S* are based on *F*^2^, conventional *R*-factors *R* are based on *F*, with *F* set to zero for negative *F*^2^. The observed criterion of *F*^2^ > σ(*F*^2^) is used only for calculating -*R*-factor-obs *etc*. and is not relevant to the choice of reflections for refinement. *R*-factors based on *F*^2^ are statistically about twice as large as those based on *F*, and *R*-factors based on ALL data will be even larger.

### Fractional atomic coordinates and isotropic or equivalent isotropic displacement parameters (Å^2^)

**Table:**

	*x*	*y*	*z*	*U*_iso_*/*U*_eq_	
N1	1.1698 (4)	−0.52205 (18)	0.12698 (19)	0.0357 (8)	
N2	1.1649 (4)	−0.54680 (16)	0.3126 (2)	0.0360 (9)	
N3	1.0216 (3)	−0.30683 (16)	0.16412 (16)	0.0294 (8)	
N4	1.0255 (3)	−0.32719 (16)	0.33648 (16)	0.0282 (7)	
C1	1.2037 (5)	−0.5982 (2)	0.1627 (3)	0.0409 (11)	
C2	1.1997 (5)	−0.6103 (2)	0.2554 (3)	0.0431 (13)	
C3	1.1299 (4)	−0.4683 (2)	0.2781 (2)	0.0296 (9)	
C4	1.1307 (4)	−0.45514 (18)	0.1822 (2)	0.0268 (8)	
C5	1.0952 (4)	−0.37680 (19)	0.1264 (2)	0.0260 (8)	
C6	1.1306 (4)	−0.3760 (2)	0.0328 (2)	0.0334 (9)	
C7	1.0901 (4)	−0.3036 (2)	−0.0181 (2)	0.0392 (10)	
C8	1.0152 (4)	−0.2326 (2)	0.0230 (2)	0.0374 (10)	
C9	0.9807 (4)	−0.2366 (2)	0.1154 (2)	0.0334 (9)	
C10	1.0970 (4)	−0.40505 (19)	0.3547 (2)	0.0280 (8)	
C11	1.1365 (4)	−0.4282 (2)	0.4450 (2)	0.0330 (10)	
C12	1.0989 (4)	−0.3714 (2)	0.5152 (2)	0.0361 (10)	
C13	1.0247 (4)	−0.2920 (2)	0.4954 (2)	0.0355 (10)	
C14	0.9907 (4)	−0.2725 (2)	0.4046 (2)	0.0332 (9)	
Au1	0.88109 (1)	0.05497 (1)	0.26548 (1)	0.0239 (1)	
Cl1	0.97121 (13)	0.19452 (5)	0.24868 (6)	0.0427 (3)	
Cl2	0.86854 (13)	0.07321 (6)	0.41954 (5)	0.0401 (3)	
Cl3	0.78564 (11)	−0.08392 (5)	0.28205 (5)	0.0346 (2)	
Cl4	0.89453 (13)	0.03479 (6)	0.11117 (5)	0.0418 (3)	
H1	1.23080	−0.64430	0.12450	0.0490*	
H2	1.22220	−0.66500	0.27880	0.0520*	
H3	0.99980	−0.30700	0.22180	0.0350*	
H6	1.18110	−0.42400	0.00480	0.0400*	
H7	1.11370	−0.30280	−0.08050	0.0470*	
H8	0.98850	−0.18330	−0.01060	0.0450*	
H9	0.92800	−0.18960	0.14430	0.0400*	
H11	1.18790	−0.48160	0.45780	0.0400*	
H12	1.12350	−0.38670	0.57550	0.0430*	
H13	0.99840	−0.25280	0.54160	0.0430*	
H14	0.94140	−0.21900	0.39040	0.0400*	

### Atomic displacement parameters (Å^2^)

**Table:**

	*U*^11^	*U*^22^	*U*^33^	*U*^12^	*U*^13^	*U*^23^
N1	0.0365 (15)	0.0331 (14)	0.0375 (15)	0.0023 (12)	0.0026 (12)	−0.0081 (12)
N2	0.0393 (15)	0.0238 (14)	0.0446 (17)	0.0003 (11)	−0.0106 (13)	0.0026 (11)
N3	0.0322 (13)	0.0321 (14)	0.0240 (12)	0.0022 (11)	0.0001 (10)	0.0000 (10)
N4	0.0301 (13)	0.0277 (13)	0.0268 (12)	0.0001 (10)	0.0003 (10)	0.0021 (10)
C1	0.0405 (19)	0.0322 (18)	0.050 (2)	0.0046 (15)	0.0005 (16)	−0.0094 (15)
C2	0.0360 (18)	0.0280 (17)	0.065 (3)	0.0023 (14)	−0.0124 (17)	−0.0007 (16)
C3	0.0251 (14)	0.0253 (14)	0.0384 (18)	−0.0014 (11)	−0.0038 (12)	0.0010 (13)
C4	0.0231 (14)	0.0268 (15)	0.0304 (15)	−0.0001 (11)	0.0011 (11)	−0.0020 (12)
C5	0.0232 (13)	0.0288 (15)	0.0260 (14)	−0.0025 (11)	−0.0014 (11)	−0.0032 (12)
C6	0.0336 (16)	0.0368 (17)	0.0299 (16)	0.0024 (13)	0.0043 (13)	−0.0030 (13)
C7	0.0327 (17)	0.055 (2)	0.0298 (16)	−0.0009 (15)	0.0047 (13)	0.0038 (15)
C8	0.0367 (17)	0.0423 (19)	0.0333 (17)	0.0008 (15)	0.0012 (13)	0.0121 (14)
C9	0.0399 (17)	0.0312 (16)	0.0289 (15)	0.0063 (13)	−0.0030 (13)	0.0006 (12)
C10	0.0272 (14)	0.0268 (15)	0.0300 (15)	−0.0050 (12)	0.0001 (12)	0.0030 (12)
C11	0.0303 (16)	0.0327 (17)	0.0359 (17)	−0.0044 (12)	−0.0045 (13)	0.0061 (13)
C12	0.0411 (18)	0.0435 (19)	0.0236 (15)	−0.0077 (15)	−0.0049 (13)	0.0024 (13)
C13	0.0385 (17)	0.0395 (18)	0.0286 (16)	−0.0011 (14)	−0.0004 (13)	−0.0044 (14)
C14	0.0368 (17)	0.0331 (16)	0.0296 (16)	0.0038 (13)	0.0032 (13)	0.0025 (13)
Au1	0.0246 (1)	0.0239 (1)	0.0232 (1)	0.0008 (1)	−0.0003 (1)	0.0022 (1)
Cl1	0.0579 (5)	0.0295 (4)	0.0407 (4)	−0.0118 (4)	−0.0047 (4)	0.0055 (3)
Cl2	0.0549 (5)	0.0410 (4)	0.0245 (4)	−0.0015 (4)	0.0016 (3)	−0.0010 (3)
Cl3	0.0420 (4)	0.0243 (3)	0.0376 (4)	−0.0005 (3)	0.0064 (3)	0.0020 (3)
Cl4	0.0535 (5)	0.0485 (5)	0.0235 (4)	−0.0127 (4)	0.0017 (3)	−0.0010 (3)

### Geometric parameters (Å, °)

**Table:**

Au1—Cl1	2.2801 (8)	C6—C7	1.380 (4)
Au1—Cl2	2.2725 (8)	C7—C8	1.374 (4)
Au1—Cl3	2.2818 (8)	C8—C9	1.378 (4)
Au1—Cl4	2.2805 (8)	C10—C11	1.397 (4)
N1—C4	1.348 (4)	C11—C12	1.383 (4)
N1—C1	1.316 (4)	C12—C13	1.379 (4)
N2—C2	1.319 (5)	C13—C14	1.383 (4)
N2—C3	1.343 (4)	C1—H1	0.9300
N3—C9	1.336 (4)	C2—H2	0.9300
N3—C5	1.336 (4)	C6—H6	0.9300
N4—C14	1.335 (4)	C7—H7	0.9300
N4—C10	1.345 (4)	C8—H8	0.9300
N3—H3	0.8600	C9—H9	0.9300
C1—C2	1.369 (6)	C11—H11	0.9300
C3—C10	1.510 (4)	C12—H12	0.9300
C3—C4	1.417 (4)	C13—H13	0.9300
C4—C5	1.487 (4)	C14—H14	0.9300
C5—C6	1.395 (4)		
			
Cl1···Cl4	3.2394 (12)	C10···N3	3.222 (4)
Cl1···C2^i^	3.471 (3)	C11···Cl4^xiv^	3.497 (3)
Cl1···Cl2	3.2230 (12)	C11···C11^ix^	3.419 (4)
Cl2···Cl3	3.2167 (12)	C11···Cl4^xii^	3.622 (3)
Cl2···Cl1	3.2230 (12)	C12···N2^ix^	3.440 (4)
Cl2···N1^ii^	3.472 (3)	C12···C8^xiv^	3.483 (4)
Cl2···C5^iii^	3.582 (3)	C12···C9^xiv^	3.592 (4)
Cl3···Cl2	3.2167 (12)	C12···Cl4^xiv^	3.627 (3)
Cl3···Cl4	3.2113 (11)	C13···C7^ii^	3.550 (4)
Cl4···Cl1	3.2394 (12)	C14···C7^ii^	3.395 (4)
Cl4···C11^iv^	3.622 (3)	C1···H7^x^	3.0500
Cl4···C12^v^	3.627 (3)	C3···H3	2.8000
Cl4···C11^v^	3.497 (3)	C7···H1^x^	2.9500
Cl4···Cl3	3.2113 (11)	C7···H14^viii^	2.9600
Cl1···H2^i^	2.9000	C9···H2^iv^	2.9000
Cl1···H7^vi^	3.0400	C10···H3	2.5700
Cl2···H6^ii^	2.9800	C12···H8^xiv^	3.0400
Cl2···H13^vii^	3.0100	C14···H3	2.7300
Cl3···H7^ii^	2.9600	C14···H1^iv^	2.9000
Cl3···H14	2.8700	C14···H7^ii^	3.0400
Cl3···H9	2.8100	H1···C14^xii^	2.9000
Cl4···H8^vi^	2.8700	H1···C7^x^	2.9500
Cl4···H12^v^	3.0900	H2···C9^xii^	2.9000
Cl4···H11^v^	2.8300	H2···Cl1^xi^	2.9000
N1···N2	2.741 (4)	H3···C3	2.8000
N1···Cl2^viii^	3.472 (3)	H3···C10	2.5700
N2···C12^ix^	3.440 (4)	H3···N4	1.7100
N2···N1	2.741 (4)	H3···C14	2.7300
N3···C10	3.222 (4)	H6···N1	2.3500
N3···N4	2.540 (3)	H6···Cl2^viii^	2.9800
N4···N3	2.540 (3)	H7···Cl1^vi^	3.0400
N4···C5	3.212 (4)	H7···C1^x^	3.0500
N1···H6	2.3500	H7···Cl3^viii^	2.9600
N2···H11	2.3600	H7···C14^viii^	3.0400
N2···H12^ix^	2.8900	H7···H14^viii^	2.4900
N4···H3	1.7100	H8···Cl4^vi^	2.8700
C1···C7^x^	3.387 (5)	H8···C12^v^	3.0400
C2···Cl1^xi^	3.471 (3)	H9···Cl3	2.8100
C2···C9^xii^	3.600 (5)	H11···N2	2.3600
C5···N4	3.212 (4)	H11···Cl4^xiv^	2.8300
C5···Cl2^xiii^	3.582 (3)	H12···Cl4^xiv^	3.0900
C7···C14^viii^	3.395 (4)	H12···N2^ix^	2.8900
C7···C1^x^	3.387 (5)	H13···Cl2^vii^	3.0100
C7···C13^viii^	3.550 (4)	H14···H7^ii^	2.4900
C8···C12^v^	3.483 (4)	H14···Cl3	2.8700
C9···C2^iv^	3.600 (5)	H14···C7^ii^	2.9600
C9···C12^v^	3.592 (4)		
			
Cl3—Au1—Cl4	89.48 (3)	C3—C10—C11	120.0 (3)
Cl1—Au1—Cl4	90.52 (3)	N4—C10—C3	120.2 (3)
Cl1—Au1—Cl2	90.14 (3)	N4—C10—C11	119.9 (3)
Cl1—Au1—Cl3	178.97 (3)	C10—C11—C12	119.7 (3)
Cl2—Au1—Cl3	89.87 (3)	C11—C12—C13	119.7 (3)
Cl2—Au1—Cl4	179.25 (3)	C12—C13—C14	117.8 (3)
C1—N1—C4	119.7 (3)	N4—C14—C13	122.8 (3)
C2—N2—C3	118.5 (3)	N1—C1—H1	120.00
C5—N3—C9	122.3 (3)	C2—C1—H1	120.00
C10—N4—C14	120.1 (2)	C1—C2—H2	119.00
C9—N3—H3	119.00	N2—C2—H2	119.00
C5—N3—H3	119.00	C5—C6—H6	120.00
N1—C1—C2	120.7 (3)	C7—C6—H6	120.00
N2—C2—C1	122.1 (3)	C6—C7—H7	120.00
C4—C3—C10	129.9 (3)	C8—C7—H7	120.00
N2—C3—C4	120.0 (3)	C9—C8—H8	121.00
N2—C3—C10	110.1 (3)	C7—C8—H8	121.00
N1—C4—C5	109.8 (3)	N3—C9—H9	119.00
N1—C4—C3	118.9 (3)	C8—C9—H9	119.00
C3—C4—C5	131.2 (3)	C10—C11—H11	120.00
C4—C5—C6	120.8 (3)	C12—C11—H11	120.00
N3—C5—C6	118.5 (3)	C13—C12—H12	120.00
N3—C5—C4	120.6 (3)	C11—C12—H12	120.00
C5—C6—C7	119.7 (3)	C12—C13—H13	121.00
C6—C7—C8	120.3 (3)	C14—C13—H13	121.00
C7—C8—C9	118.1 (3)	C13—C14—H14	119.00
N3—C9—C8	121.1 (3)	N4—C14—H14	119.00
			
C4—N1—C1—C2	0.0 (5)	N2—C3—C10—C11	11.3 (4)
C1—N1—C4—C3	1.0 (5)	C4—C3—C10—N4	14.0 (5)
C1—N1—C4—C5	−179.2 (3)	C4—C3—C10—C11	−167.7 (3)
C3—N2—C2—C1	1.0 (5)	N1—C4—C5—N3	167.6 (3)
C2—N2—C3—C4	0.1 (5)	N1—C4—C5—C6	−9.6 (4)
C2—N2—C3—C10	−179.1 (3)	C3—C4—C5—N3	−12.7 (5)
C9—N3—C5—C4	−177.1 (3)	C3—C4—C5—C6	170.2 (3)
C9—N3—C5—C6	0.1 (4)	N3—C5—C6—C7	0.3 (4)
C5—N3—C9—C8	−0.9 (4)	C4—C5—C6—C7	177.6 (3)
C14—N4—C10—C3	177.7 (3)	C5—C6—C7—C8	0.0 (4)
C14—N4—C10—C11	−0.7 (4)	C6—C7—C8—C9	−0.7 (4)
C10—N4—C14—C13	−0.1 (4)	C7—C8—C9—N3	1.1 (4)
N1—C1—C2—N2	−1.1 (6)	N4—C10—C11—C12	1.1 (4)
N2—C3—C4—N1	−1.1 (4)	C3—C10—C11—C12	−177.3 (3)
N2—C3—C4—C5	179.2 (3)	C10—C11—C12—C13	−0.8 (4)
C10—C3—C4—N1	177.9 (3)	C11—C12—C13—C14	0.0 (4)
C10—C3—C4—C5	−1.9 (5)	C12—C13—C14—N4	0.5 (5)
N2—C3—C10—N4	−167.1 (3)		

Symmetry codes: (i) *x*, *y*+1, *z*; (ii) *x*−1/2, −*y*−1/2, *z*+1/2; (iii) −*x*+3/2, *y*+1/2, −*z*+1/2; (iv) −*x*+5/2, *y*+1/2, −*z*+1/2; (v) *x*−1/2, −*y*−1/2, *z*−1/2; (vi) −*x*+2, −*y*, −*z*; (vii) −*x*+2, −*y*, −*z*+1; (viii) *x*+1/2, −*y*−1/2, *z*−1/2; (ix) −*x*+2, −*y*−1, −*z*+1; (x) −*x*+2, −*y*−1, −*z*; (xi) *x*, *y*−1, *z*; (xii) −*x*+5/2, *y*−1/2, −*z*+1/2; (xiii) −*x*+3/2, *y*−1/2, −*z*+1/2; (xiv) *x*+1/2, −*y*−1/2, *z*+1/2.

### Hydrogen-bond geometry (Å, °)

**Table:**

*D*—H···*A*	*D*—H	H···*A*	*D*···*A*	*D*—H···*A*
N3—H3···N4	0.86	1.71	2.540 (3)	160
C6—H6···N1	0.93	2.35	2.667 (4)	100
C9—H9···Cl3	0.93	2.81	3.699 (3)	161
C11—H11···N2	0.93	2.36	2.680 (4)	100
C11—H11···Cl4^xiv^	0.93	2.83	3.497 (3)	130

Symmetry codes: (xiv) *x*+1/2, −*y*−1/2, *z*+1/2.
